# Porous Graphene Oxide Prepared on Nickel Foam by Electrophoretic Deposition and Thermal Reduction as High-Performance Supercapacitor Electrodes

**DOI:** 10.3390/ma10080936

**Published:** 2017-08-11

**Authors:** Yunhe Xu, Jun Li, Wenxin Huang

**Affiliations:** School of Materials Engineering, Shanghai University of Engineering Science, Shanghai 201620, China; zgsdxyh@sina.com (Y.X.); vensin_huang@163.com (W.H.)

**Keywords:** graphene oxide, electrophoretic deposition, thermal reduction, supercapacitor, electrochemical performance

## Abstract

A simple electrophoretic deposition method was developed to prepare graphene oxide (GO) films on the frameworks of nickel foam without any conductive agents and polymer binders. Then, GO was transformed into thermally-reduced graphene oxide (RGO) at an appropriate temperature. The effects of deposition voltage and thermal reduction temperature on the electrochemical properties of RGO were investigated by cyclic voltammetry (CV) and galvanostatic charge/discharge. The appropriate combination of deposition voltage and thermal reduction temperature was established. Moreover, scanning electron microscopy, thermal gravimetric analysis, differential thermal analysis, Fourier transform infrared spectroscopy, Raman spectroscopy, and X-ray diffractometry were applied to validate the results, which showed that the highest specific capacitance of RGO was obtained when the deposition voltage was 60 V and the thermal reduction temperature was 300 °C. The specific capacitance values calculated by CV and galvanostatic charge/discharge were 139 F·g^−1^ (0.005 V·s^−1^) and 151 F·g^−1^ (1 A·g^−1^), respectively. The specific capacitance of RGO maintained 55% and 66% of the initial value when the scan rate and the current density were increased up to 0.3 V·s^−1^ and 10 A·g^−1^, respectively. RGO also displayed an excellent cycling stability by maintaining 98% of the initial specific capacitance after 500 cycles.

## 1. Introduction

Energy scarcity is one of the serious challenges of the 21st century; the depletion of fossil fuel resources and the worsening environmental problems create an urgent demand for environmentally-friendly high-power energy storage devices to satisfy the power demands [1,2]. Supercapacitors have recently attracted significant attention because they can operate at high charge/discharge rates over an almost unlimited number of cycles, enable energy recovery in heavy-duty systems, and are relatively inexpensive [3].

The electrode material is the most important factor in the supercapacitor, which directly affects the properties of the supercapacitor. The electrode material is usually divided into three kinds, corresponding to carbon material, conductive polymer, and metallic oxide. Among them, carbon material is regarded as the most promising electrode material applied in industry. The carbon material includes activated carbon, porous carbon, carbon nonotubes, and grapheme [4]. Activated carbon can be prepared by the activation method with some materials (such as mesophase pitch, poly, sucrose, and so on) as the precursors. The activation agents include KOH [5], NaOH [6], CO_2_ [7], and so on. The activated carbons (ACs) prepared from mesophase pitch with KOH etching were used to fabricate electrodes for electric double layer capacitors (EDLC) [5]. The performance of the ACs in the organic solvent (1 M of Et_4_NBF_4_ in propylene carbonate) was investigated with voltage sweep cyclic voltammetry and constant-current charge-discharge cycling. The sample with a surface area of 2258 m^2^·g^−1^ possessed a specific capacitance as high as 145 F·g^−1^. Xu et al. [6] prepared mesoporous activated carbons with high surface area by NaOH activation for non-aqueous EDLC. The material with optimized pore size presented both a high capacitance of 155 F·g^−1^ and outstanding rate capability in non-aqueous electrolytes. As the current density was increased to 18,000 mA·g^−1^, it remained 109 F·g^−1^, an attractive value for EDLC. 

Lu et al. [7] prepared activated sucrose-derived carbons (ASCs) by the pyrolysis of sucrose followed by the activation with CO_2_ gas for 1–5 h at 900 °C. The material exhibited a comparatively high specific capacitance of about 160 F·g^−1^ and an excellent frequency response in a two-electrode EDLC cell with 1 M H_2_SO_4_ as the electrolyte. Although activated carbon is most widely used in EDLC, there are two inherent shortcomings corresponding to low specific capacitance at a high current and poor rate capacity performance. Porous carbon is also a promising electrode material used in the supercapacitor, which is usually produced by the template method. 

Liu et al. [8] prepared the ordered hierarchical mesoporous/microporous carbon nanomaterial (OHMMC) derived from mesoporous titanium-carbide/carbon composites by the template method. The material exhibited a hexagonal mesostructure of 3 nm, around which a large number of microporous with the size ranging from 0.6 to 1.3 nm were observed. The unique structure endowed the material with excellent electrochemical properties. Its specific capacitance reached 146 F·g^−1^ at a current density of 100 mA·g^−1^ in 1 M (C_2_H_5_)_4_NBF_4_/PC electrolyte. Moreover, it also exhibited the excellent cycle performance (83% of its initial capacity was maintained after 5000 cycles). Liu et al. [9] synthesized the order mesoporous carbon nanofiber arrays (MCNAs) from a crab shell biological template. The arrays with a very high specific area (1266 m^2^·g^−1^) included a larger number of highly-ordered mesopores with diameters of about 11.5 nm. The specific capacitance of MCNAs was 152 F·g^−1^ at a current density of 100 mA·g^−1^ in 1 M (C_2_H_5_)_4_NBF_4_/PC electrolyte. 

Huang et al. [10] directly synthesized three-dimensional, hierarchically ordered, porous carbon (HOPC) by means of a simple one-pot synthesis procedure. The designed porous textures of the 3D HOPC materials were composed of highly-ordered, fcc macropores (300 nm), interconnected porous structures, including macroporous windows (170 nm), hexagonally-ordered mesopores (5.0 nm), and useful micropores (1.2 nm). 3D HOPC-g-1000 (g = graphitic, 1000 = pyrolysis temperature of 1000 °C) with partially graphitic nanostructures had a low gravimetric specific capacitance (73.4 F·g^−1^ at 3 mV·s^−1^), but a better rate performance and excellent cycling performance (>5400 cycles). It can be seen that porous carbon with the anticipated structure and physicochemical properties can be produced by selecting the suitable template, carbon source, and processing parameters. However, a few issues need to be overcome (such as high cost, low productivity, complicated preparation process, and so on). Carbon nanotubes are widely concerned about due to their unique structure, high electroconductivity, excellent mechanical properties, and good chemical/thermal stability. Those advantages are what the supercapacitor needs. An et al. [11] synthesized single-walled CNTs by DC-arc discharge under a helium pressure of 13,332 Pa, and investigated the key factors determining the performance of supercapacitors using single-walled CNTs as the electrode. A maximum specific capacitance of 180 F·g^−1^ and a large power density of 20 kW·kg^−1^ were obtained at an energy density of 6.5 Wh·kg^−1^ in 7.5 N KOH aqueous solution. Hiraoka et al. [12] fabricated the carbon nanotubes with a very high specific area of 2240 m^2^·g^−1^ by opening single-walled carbon nanotubes (SWNT) forests via controlled oxidation. The specific capacitance reached 114 F·g^−1^ in a tetraethylammonium tetrafluoroborate (Et_4_NBF_4_)/propylene. Although the investigations into carbon nanotubes as the electrode material of the supercapacitor have been carried out, the complicated preparation process, high cost, and comparatively low specific area still seriously limit their application in industry.

Graphene has been considered an ideal electrode material for supercapacitors because of its outstanding conductivity, superior theoretical capacitance, high theoretical surface area, and electrochemical stability [13]. Many routes have been developed so far to prepare graphene for supercapacitors [14,15]. One of the promising routes for the large-scale and low-cost preparation of graphene is the reduction of graphene oxide (GO) [16,17,18], in which GO is first prepared by oxidizing graphite, which is subsequently converted into GO by chemical exfoliation [19]. GO can be viewed as a graphene layer that is asymmetrically decorated with oxygen-containing functional groups on the basal plane and along the edges [20]. Finally, GO is further transformed into reduced graphene oxide (RGO) by chemical or thermal reduction. Chemical reduction is effective for producing graphene. However, the strong reducing agents (such as hydrazine) used in this method are hazardous and toxic [21,22]. Consequently, several chemical reduction systems composed of safe and environmentally-friendly agents have been explored to produce graphene. Liu et al. [23] fabricated RGO by solvothermal synthesis. In this method, a reaction system composed of GO (60 mg) and ethanol (60 mL) is heated at temperatures that range from 413 to 493 K for 10 h. The product is dried in vacuum at 323 K for 24 h. Their results revealed that only the carbonyl group is retained. Tao et al. [24] also prepared RGO by solvothermal synthesis. In their method, GO is dispersed in deionized water and heated in an autoclave at 180 °C for 12 h to obtain reduced GO hydrogels. Then, the hydrogels are freeze-dried to obtain the corresponding reduced GO foams. The agents used in these methods are clean and green. However, they are complicated, time-consuming, and require high temperatures.

Thermal reduction was recently introduced as an efficient method of obtaining graphene [25]. The thermal reduction of GO is more convenient than chemical reduction and does not use hazardous reductants. Xia et al. [26] prepared RGO through the thermal reduction of GO at 850 °C for 6 h. RGO was mixed with acetylene black and polytetrafluoroethylene at a mass ratio of 8:1:1. The electrode was prepared by pressing the mixture onto a 1 cm^2^ nickel foam at 10 MPa and dried at 70 °C overnight. Li et al. [27] also prepared RGO by thermal reduction. They rapidly heated GO at 800 °C for 30 s to obtain RGO. RGO, SnO_2_ nanoplatelets, carbon black, and poly were mixed and dispersed in the ethanol solution to obtain the slurry, which was coated onto a nickel foam to form an electrode. Xiao et al. [28] prepared RGO through the thermal reduction of GO at 300 °C for 3 h. They assembled the electrode by coating a nickel foam with a slurry consisting of RGO, acetylene black, conducting graphite, and ethanol. Notably, RGO is usually prepared in powder form, which needs to be mixed with additives to form an electrode [29,30,31]. However, additives may decrease the conductivity of the electrode. Moreover, manual smearing methods, such as press slice [32], spray-coating [33], and spin coating [34], are usually used to assemble the mixture and the substrate into an electrode, thereby making the electrochemical properties of the electrode difficult to control precisely. Therefore, a new method of preparing RGO-based electrodes with good reproducibility and without additives should be sought.

Electrophoretic deposition (EPD) is regarded an economical and versatile alternative of preparing GO films with controllable thickness and morphology on different substrates [35,36]. Wang et al. [37] directly deposited GO on carbon fibers (CFs) by EPD with an applied voltage of 160 V in isopropyl alcohol as the electrolytic solution. Their results indicated that a thin layer of GO sheet with a wrinkled structure was successfully deposited on the CF. Ghasemi et al. [38] prepared a thin GO film on stainless steel (SS) by EPD from an aqueous dispersion of GO with an applied voltage of 4 V for 5 min. The film adhered to the SS without peeling off from the surface. Xiong et al. [39] studied the deposition of GO on nickel foam by EPD. Their reaction system was composed of GO (200 mg) and ethanol (100 mL), and the deposition was conducted for 1 min at a voltage of 2 to 5 V. After EPD, the substrate covered with GO is thermally reduced at high temperatures [40,41], during which GO is thermally reduced. Therefore, GO-based electrodes are prepared by EPD followed by thermal reduction.

However, the effects of deposition voltage and thermal reduction temperature on the electrochemical properties of RGO electrodes have not been investigated in detail. Deposition voltage is an important parameter of EPD because it directly affects the thickness and morphology of deposits. A low voltage may result in the incomplete covering of the substrate, thereby reducing the stored energy of the RGO-based supercapacitor significantly. On the contrary, a high voltage may lead to the control of deposition by the diffusion of GO particles, which not only reduces the deposition rate, but also deteriorates the quality of the deposited GO. Thermal reduction temperature is also an important factor in the final electrochemical properties of RGO. A high temperature contributes to enhancing the conductivity and the surface area of RGO by the exfoliation of RGO and the removal of oxygen-containing groups in GO, thereby improving the EDLC of RGO [40]. However, the pseudocapacitance contribution may be eliminated due to the complete removal of the oxygen-containing groups. The analyses above indicate that an RGO prepared with the optimum combination of deposition voltage and thermal reduction temperature can exhibit the best electrochemical properties.

In this paper, GO was deposited on nickel foam by EPD in an aqueous dispersion of GO, and then thermally reduced to RGO at high temperatures. The effects of deposition voltage and thermal reduction temperature on the electrochemical performance of RGO electrodes were studied systematically. The appropriate combination of the deposition voltage and thermal reduction temperature was established.

## 2. Experimental

Nickel foam with high specific surface area can effectively prevent the stacking and agglomeration of GO particles and carry many active substances (GO), which are endowed with a high specific capacitance [42]. As the anode, commercial nickel foam (20 mm × 25 mm, Shanxi Power Source Battery Materials Co., Ltd., Taiyuan, Shangxi, China) was treated in 0.1 mol·L^−1^ in diluted hydrochloric acid by ultrasonic waves for 30 min and cleaned with deionized water, ethanol, and acetone. Then, the nickel foam was weighed using a BSA124S electronic balance (0.1 mg, Sartorious, Beijing, China). Flaky graphite (20 mm × 25 mm, Beijing Electrical Carbon Factory, Beijing, China) was chosen as the cathode. GO (50 mg), prepared through a modified Hummers method, [43] was mixed with 100 mL of deionized water, and then processed for 2 h by ultrasonic waves to yield a stable GO suspension (0.5 mg·mL^−1^) as the electrolyte.

**Figure 1:**
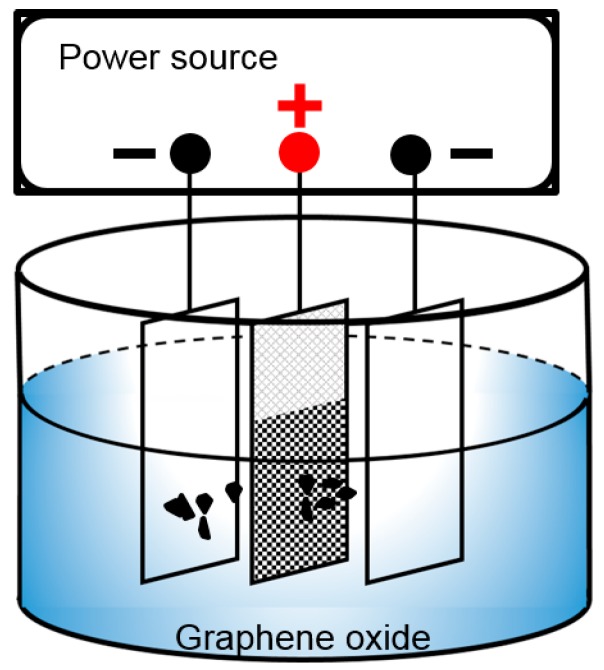
Schematic diagram of the EPD process.

The schematic of EPD is depicted in Figure 1. The GO suspension was poured into an electrocoat tank. Two graphite pieces were symmetrically placed on both sides of a piece of nickel foam, between which 1 cm was maintained. The applied deposition voltages were 20, 40, 60, 80, and 100 V. The anode was naturally air-dried after deposition. Then, the GO deposited on the nickel foam was transformed into RGO in a muffle stove in an argon atmosphere at 200, 300, and 400 °C for 2 h. The electrode was weighed again to obtain the weight of RGO by subtracting the weight of the nickel foam.

The morphology of GO before and after thermal reduction was examined using a S-3400 scanning electron microscope (SEM, Hitachi , Tokyo, Japan). The thermal reduction properties of GO were characterized by an SDT Q600 V20.9 Build 20 thermal gravimetric and differential thermal analyzer (TGA–DTA, TA Instruments, New Castle, DE, USA), a Nicolet FT-IR370 (Thermo Fisher Scientific, Waltham, MA, USA) Fourier transform infrared spectrometer (FTIR), and a Raman spectrometer (Renishaw, Dongguan, Guangdong, China). Phase constituents of nickel foam before and after treatment (Electrophoretic deposition and thermal reduction) were identified using a X’ Pert Pro X-ray diffractometer (XRD, PANalytical, Eindhoven, ZuidHolland, The Netherlands) with Cu Kα radiation (λ = 0.1540560 nm).The electrochemical performance was measured by cyclic voltammetry (CV) and galvanostatic charge/discharge on a CHI 6082D (CH Instruments Ins, Shanghai, China) electrochemical workstation with the nickel foam covered with RGO as the working electrode, the graphite sheet as the counter electrode, and the saturated calomel electrode (SCE) as the reference. A 0.5 M Na_2_SO_4_ aqueous solution was selected as the electrolyte. The CV scans were recorded from −0.2 to 0.8 V (vs. SCE) at different scan rates (0.005, 0.01, 0.05, 0.1, and 0.3 V·s^−1^). Galvanostatic charge/discharge was performed in the potential range of −0.2 to 0.8 V at different constant current densities (1, 1.5, 3, 5, and 10 A·g^−1^).

## 3. Results and Discussion

### 3.1. Morphological Characterization of GO

**Figure 2:**
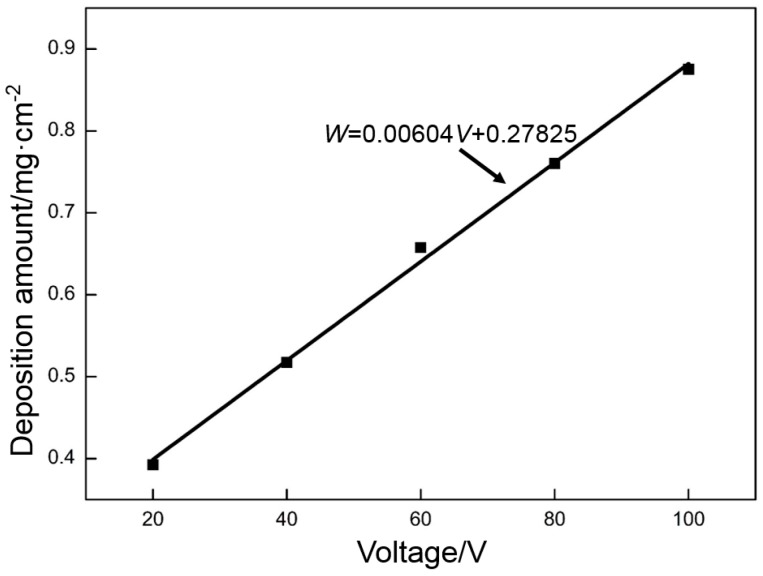
Relationship between voltage and deposition amount.

Figure 2 show the relationship curve between the voltage and deposition amount of GO. Both approximately present a linear relationship. These data were processed by the least squares method, and the fitting result is as follows:

*W* = 0.00604*V* + 0.27825 (1)

where *W* is the deposition amount (mg·cm^−2^) and *V* is the voltage (V).

The resistance is approximately constant in the electrodeposition system. According to Ohm’s law, a high voltage causes many GO particles to move and deposit on the nickel foam. This law is consistent with the experimental result. However, this agreement does not mean that a better deposition quality can be obtained with a higher deposition voltage.

**Figure 3:**
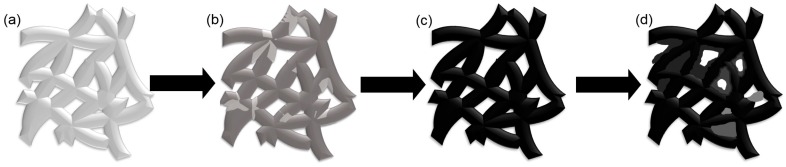
Schematic drawing of morphological evolution of GO at different deposition voltages (**a**) nickel foam; (**b**) GO was deposited on nickel foam at 40 V; (**c**) GO was deposited on nickel foam at 60 V; (**d**) GO was deposited on nickel foam at 80 V.

The above analyses indicate that a layer of high-quality GO can be obtained at a deposition voltage of 60 V. Figure 3 shows the morphological evolution of the nickel foam with the change in deposition voltage. When a low deposition voltage (20 and 40 V) is used, some frameworks in the nickel foam are not completely covered with GO (Figure 3b). The naked zones without active substances lead to the loss of specific capacitance. However, when an extremely high deposition voltage is applied (80 and 100 V), some GO particles may aggregate on the edges of the frameworks, spread around, and finally cover some holes around the frameworks (Figure 3d). This activity prevents ions from migrating toward the inner part of the nickel foam, thereby decreasing the specific capacitance. Obviously, the nickel foam with all frameworks tightly covered with a dense layer of GO (60 V) exhibits the best performance (Figure 3c). This finding is further confirmed by SEM.

**Figure 4:**
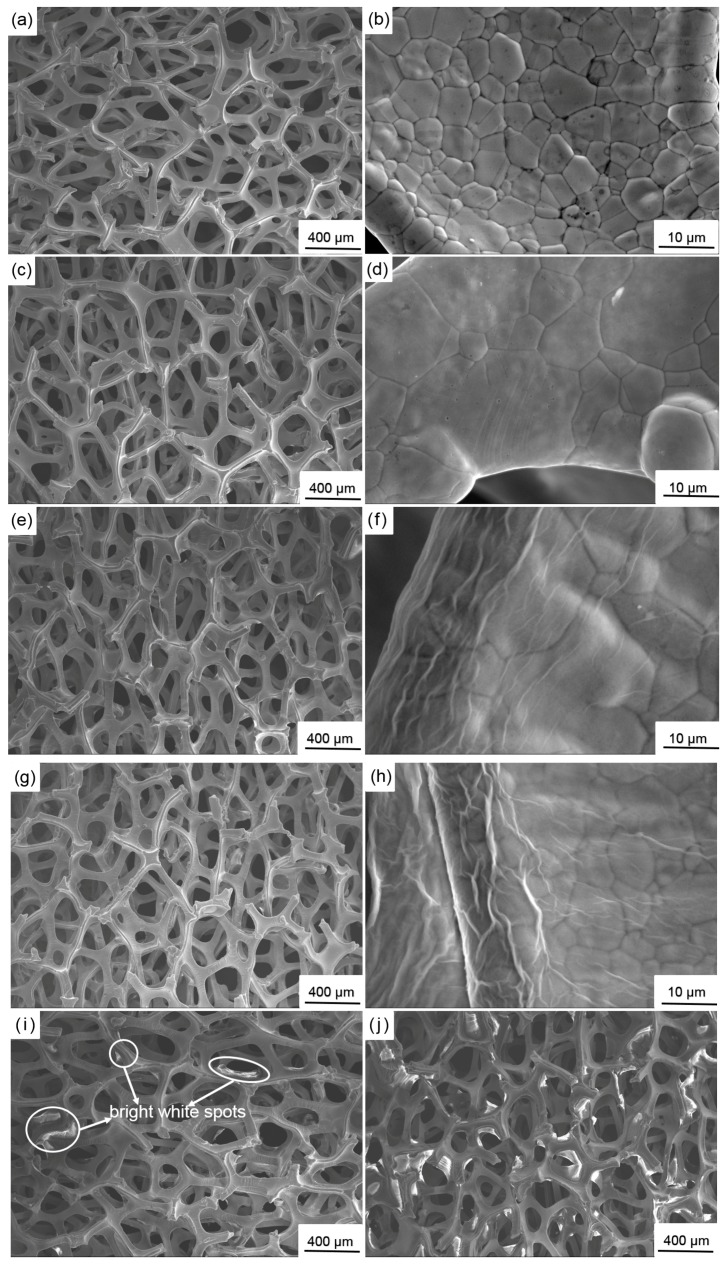
SEM images of (**a**,**b**) initial nickel foam and nickel foam covered with GO deposited at different voltages :(**c**,**d**) 20 V; (**e**,**f**) 40 V; (**g**,**h**) 60 V; (**i**) 80 V; and (**j**) 100 V.

Figure 4a shows that the initial nickel foam is composed of numerous three-dimensional, cross-linked frameworks, among which many pores can be clearly observed. After deposition at 20, 40, and 60 V, the nickel foam presents a similar structure as shown in Figure 4c,e,g in low-magnification SEM images. However, the high-magnification SEM images clearly reveal a regular change before and after deposition. The framework consists of equiaxial grains and the grain boundary is clearly visible prior to deposition. The grain boundary becomes obscure after deposition at 20 V, thereby indicating that the framework is covered with a thin layer of GO. Several fold-like patterns are formed around the edges of the frameworks at 40 V. These patterns become more obvious at 60 V. This phenomenon is relevant to the edge effect. The charge density around the electrode edge is higher than that in the other zones and proportional to the electric field strength. Therefore, more GO particles tend to be deposited around the electrode edge the stronger the electric field. The deposited GO on the electrode edge flows slowly under gravity when the electrode is removed from the GO dispersion, thereby resulting in the formation of fold-like patterns that are approximately parallel to the direction of gravity. The morphology of GO significantly changes at 80 V. Some bright white blocky and strip-shaped products appear on the edges of the frameworks (Figure 4i). When the deposition voltage is further increased to 100 V similar, but larger, products are visible and some holes around the frameworks are covered (Figure 4j). These products may be the over-deposited GO. When GO is radiated by secondary electrons in the SEM observation, numerous electrons aggregate on the surface of GO due to its poor electrical conductivity, thereby resulting in the formation of white zones [44].

### 3.2. Thermal Reduction Properties of GO

**Figure 5:**
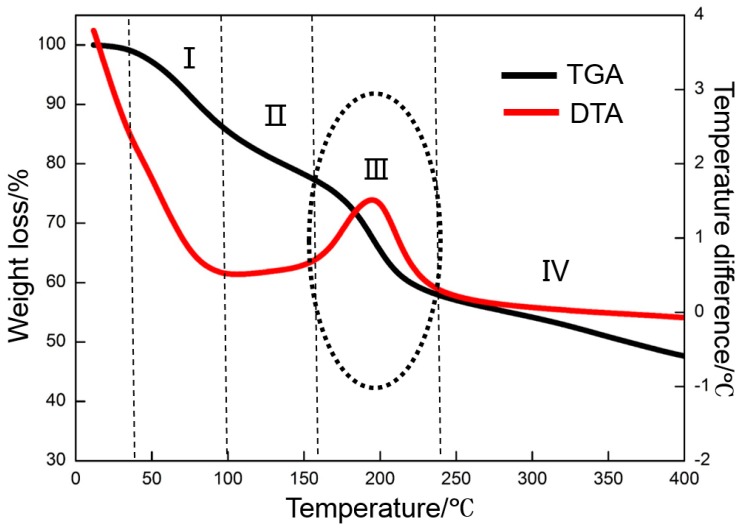
TGA-DTA curves of GO in a nitrogen atmosphere.

GO cannot store electrons as an insulated material [45]. Therefore, the deposited GO must be reduced to graphene with a strong ability to store numerous electrons at a certain temperature. The oxygen-containing functional groups in GO cannot be removed efficiently when the temperature is extremely low. However, a high temperature may burn the nickel foam. Therefore, an appropriate thermal reduction temperature should be selected. The appropriate temperature is confirmed by TGA and DTA tests (Figure 5). The TGA curve shows that the weight loss of GO can be divided into four stages as marked in Figure 5. The weight loss is negligible when the temperature is below 40 °C. In the first stage, whose temperature ranges from 40 to 100 °C, the weight loss is approximately 15%, which is ascribed to the evaporation of physically-absorbed water in GO. As the temperature increases from 100 to 160 °C (the second stage), the slope of the curve is lower than that at the first stage, thereby suggesting a decreasing weight loss rate. The weight loss of approximately 9% at this stage mainly results from the evaporation of chemically-absorbed water in the GO. When the temperature exceeds 160 °C, the weight loss is suddenly accelerated, accompanied with an abrupt weight loss of 16% in a narrow temperature range of 160 to 240 °C. The weight loss at this stage is mainly caused by the removal of numerous labile oxygen-containing functional groups in GO, which is consistent with previous studies [46]. Slightly more weight is lost with the further increase in temperature due to some gasification reactions that occur between carbon and the impurities (such as oxygen) in N_2_ [47]. The DTA result is consistent with that of TGA. A marked exothermic DTA signal is observed at the third stage. The TGA and DTA test results show that the oxygen-containing functional groups of GO decomposition are decomposed at approximately 160 °C and decomposition is completed at approximately 240 °C. Therefore, 300 °C is selected as the thermal reduction temperature, and 200 and 400 °C are selected as references.

**Figure 6:**
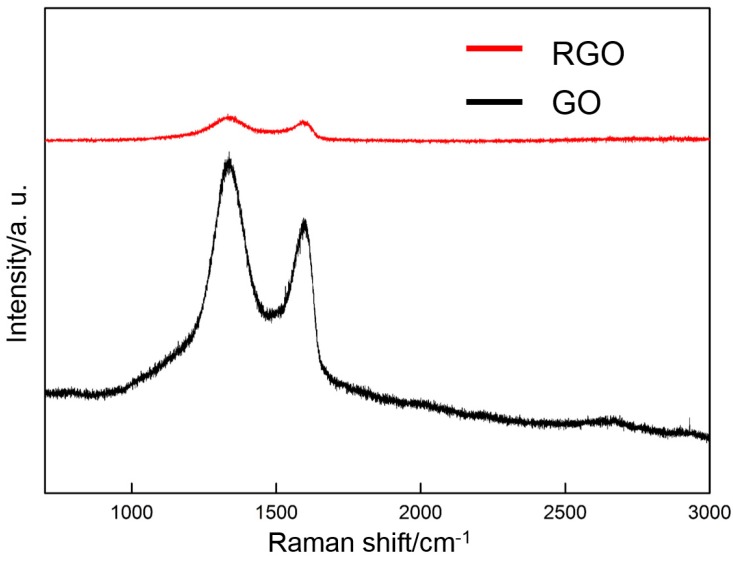
Raman spectra of GO and RGO after thermal reduction (300 °C).

Raman spectroscopy is used to characterize the change in the molecular structure of the deposited GO (60 V) before and after thermal reduction (300 °C). Figure 6 shows that three distinct characteristic peaks can be observed in the Raman spectrum of the deposited GO before thermal reduction: the D-band, G-band, and broad 2D-band around 1337, 1595, and 2700 cm^−1^, respectively. The D-band is due to the defects or edges [48], and the G-band corresponds to the E_2g_ phonon at the Brillouin zone [49]. After thermal reduction, the D-band and the G-band are shifted to 1340 and 1607 cm^−1^, respectively, thereby indicating the reduction of GO by thermal treatment.

**Figure 7:**
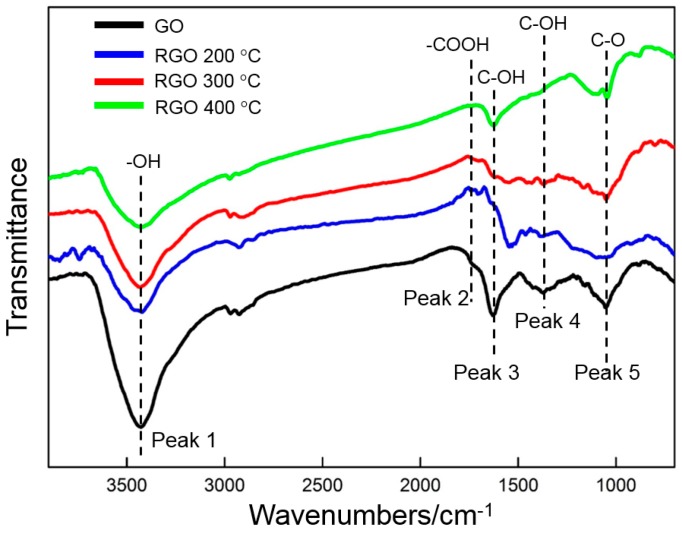
FTIR curves of GO before and after thermal reduction at different temperatures.

FTIR is used to analyze the oxygen-containing functional groups of GO before and after thermal reduction at different temperatures (Figure 7). Five oxygen-containing functional groups can be clearly identified prior to thermal reduction. A strong and broad absorption peak (peak 1) is at approximately 3427 cm^−1^, which can be attributed to the stretching vibration of –OH in water and GO [32]. The weak peak (peak 2) at 1730 cm^−1^ is related to the stretching vibrations of C=O in the carboxyl and carbonyl groups. The two strong peaks (peaks 3 and 4) at 1630 and 1371 cm^−1^ result from the bending vibration of –OH in the carboxyl groups. The C–O epoxide group can be identified by the peak (peak 5) at 1054 cm^−1^. The above result confirms that many oxygen-containing functional groups (hydroxyl, carboxyl, carbonyl, and epoxide) exist in the deposited GO, which has also been verified by several studies [15,22]. After the thermal reduction at 200 °C, only the intensity of peak 1, which is related to –OH, decreases obviously. The other peaks only slightly decrease in intensity. Those characteristic peaks change after thermal reduction at 300 °C. The intensity of the three peaks that are related to –OH (peaks 1, 3, and 4) decreases, and those of the latter two are nearly invisible. This finding indicates that hydroxyl groups can be removed efficiently by thermal reduction. Moreover, the two peaks at 1730 and 1054 cm^−1^ (peaks 2 and 5) also become weak. The intensity of all peaks is further reduced by the thermal treatment at 400 °C. The FTIR result shows that GO can be reduced by thermal treatment.

### 3.3. XRD Results

**Figure 8:**
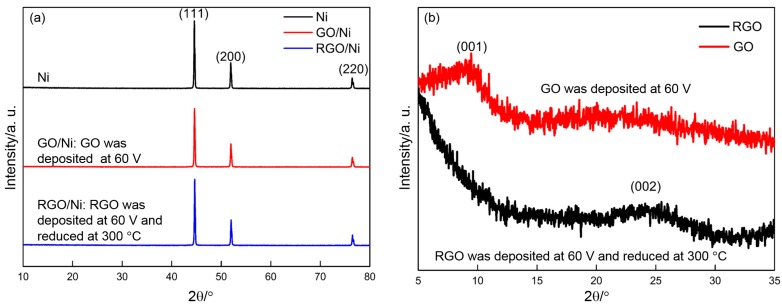
(**a**) XRD patterns of Ni, GO/Ni, and RGO/Ni; and (**b**) XRD patterns of GO and RGO.

Figure 8a shows XRD patterns of Ni, GO/Ni, and RGO/Ni. Their patterns are highly similar, which indicates that their phase constituents are same. Three sharp peaks are located at 2θ = 44.6°, 2θ = 51.9°, and 2θ = 76.4°, which are in accordance with the JCPDS card (No. 04-0850 for Ni). This illustrates that phase constituents of nickel foam are unchanged after deposition and thermal reduction. Due to deposited GO on nickel foam is very litter, no GO and RGO are observed from Figure 8a. In order to identify GO and RGO clearly, sufficient GO was scraped off from many pieces of GO/Ni electrodes (prepared at 60 V) and collected for XRD analyses. Then GO powder was thermally reduced at 300 °C and further identified by XRD. Figure 8b displays XRD patterns of GO and RGO powder. For the powder deposited at 60 V, a board (001) diffraction peak is observed clearly at about 2θ = 10°, indicating that the existence of GO [35]. After thermal reduction at 300 °C, the diffraction peak (2θ = 10°) related to GO disappears and a broader and weaker (002) peak arises near 24° [38], suggesting the reduction of GO [50].

### 3.4. Electrochemical Properties of RGO

**Figure 9:**
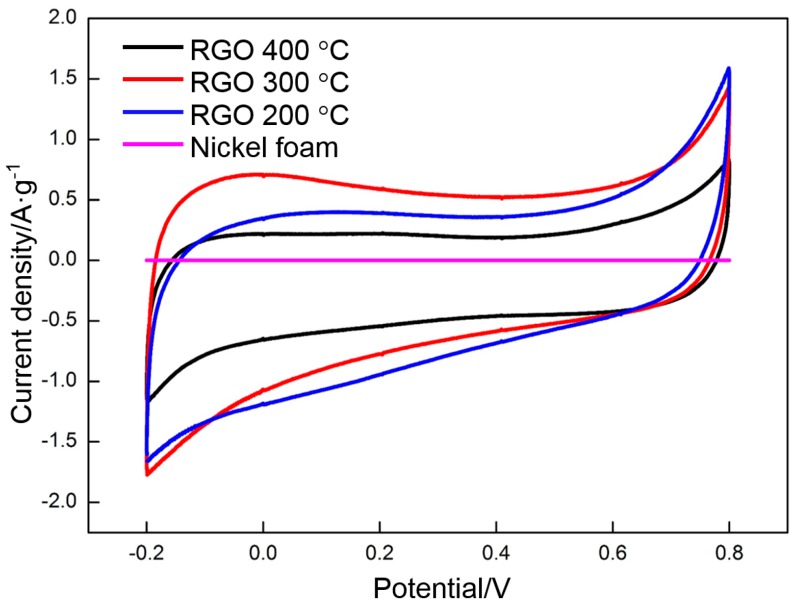
CV curves of nickel foam and RGO reduced at different temperatures.

GO was deposited at 60 V and reduced at 200, 300, and 400 °C. The effect of reduction temperature on the specific capacitance of RGO is assessed by CV. Figure 9 shows the CV profiles of RGO (200, 300, and 400 °C) and nickel foam at a scan rate of 0.005 V·s^−1^ in the potential range of −0.2 V to 0.8 V. The CV curve of nickel foam presents an approximate line. The integrated area of the profile is negligible, thereby indicating a small capacitance. The shape of the CV curves significantly changes when GO is deposited on the nickel foam and undergoes thermal reduction at different temperatures. A rectangular shape with a large integrated area is observed and indicates that RGO possesses a high capacitance. The specific capacitance of RGO can be calculated according to the following equation:

(2) $$C_{m}=\frac{\int idV}{2\times m\times \Delta V\times S}$$

in which *C_m_* is the specific capacitance measured from CV tests (F·g^−1^), ∫idV is the integrated area of CV curves, *m* is the mass of RGO (g), ΔV is the potential range (V), and S is the scan rate (V·s^−1^).

The integrated area of the nickel foam curve in Figure 9 is subtracted to obtain an accurate specific capacitance of RGO. The specific capacitance values of RGO (200, 300, and 400 °C) before and after subtraction are 105, 139, and 98 F·g^−1^, respectively. Therefore, the effect of the nickel foam on specific capacitance is negligible. The thermal reduction temperature has a significant effect on the specific capacitance of RGO. The CV curve of RGO thermally reduced at 400 °C has a nearly rectangular shape with the smallest area, thereby indicating that its capacitance mainly comes from EDLC. The contribution of pseudocapacitance to the specific capacitance is nearly negligible. The CV curve of RGO begins to deviate from the rectangular shape and the specific capacitance is significantly increased when the temperature is reduced to 300 °C. The change is closely related to the oxygen-containing functional groups in the RGO. More groups in the RGO can be maintained at 300 °C than at higher temperatures, thereby not only increasing the surface wettability and accessible electroactive surface area, but also providing high pseudocapacitance than in that thermally reduced at 400 °C [25]. Pseudocapacitance mainly comes from the reversible redox reactions among the hydroxyl groups, namely, carbonyl, carboxyl, and lactone groups. These redox reactions can be expressed as follows [51]:

(3) $$>C-\mathrm{OH}=\Leftrightarrow >C=O+H^{+}+e^{-}$$

(4) $$-\mathrm{COOH}\Leftrightarrow -\mathrm{COO}+H^{+}+e^{-}$$

(5) $$>C=O+e^{-}\Leftrightarrow >C-O^{-}$$

With the further decrease in temperature to 200 °C, the above reactions should occur more easily due to the existence of more oxygen-containing functional groups than at higher temperatures. The capacitance should be improved correspondingly. However, this is not the case. The capacitance is lower than that of the RGO that is thermally reduced at 300 °C. Pseudocapacitance depends on the redox reactions in RGO. However, the EDLC mainly results from the free electrons in RGO. Several studies have investigated the effect of temperature on the conductivity of RGO. The results indicated that the conductivity of RGO (200 °C) is poor, thereby indicating that few electrons exist in RGO (200 °C). Therefore, the decrease in the capacitance of RGO (200 °C) should be attributed to the dramatic reduction in the number of free electrons.

**Figure 10:**
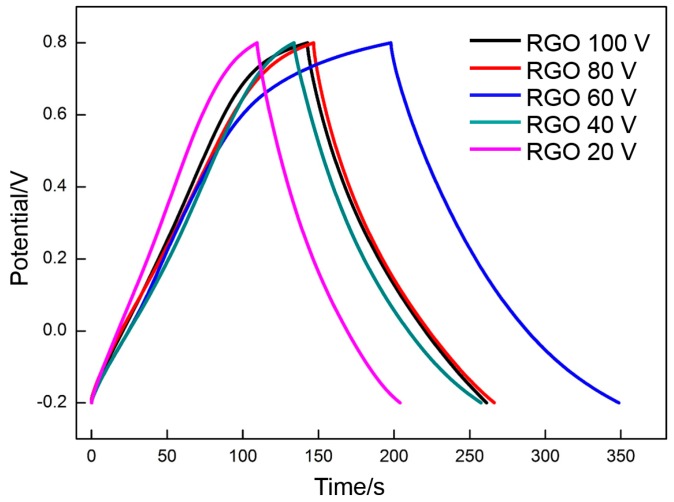
Galvanostatic charge/discharge curves of RGO reduced at different temperatures.

The galvanostatic charge/discharge curves of RGO (200, 300, and 400 °C) are shown in Figure 10. The curve of the RGO that is thermally reduced at 400 °C exhibits a typical symmetrical triangular shape, thereby demonstrating the EDLC characteristic. The other curves (especially that of the RGO that is thermally reduced at 300 °C) present a nonlinear pseudocapacitive characteristic, and obvious oxidation reduction peaks can be clearly observed. The specific capacitance of RGO can be calculated according to the following equation:

(6) $$C_{m}=\frac{I\cdot \Delta t}{m\cdot \Delta v}$$

where *C_m_* is the specific capacitance measured from galvanostatic charge/discharge curve (F·g^−1^), *I* represents the constant discharge current (A), *m* is the mass of RGO (g), and Δv is the potential range (V).

The specific capacitance values of the RGO thermally reduced at 200, 300, and 400 °C are 118, 151, and 92 F·g^−1^, respectively. The results are similar to those measured by CV, thereby further illustrating that 300 °C is the appropriate reduction temperature.

**Figure 11:**
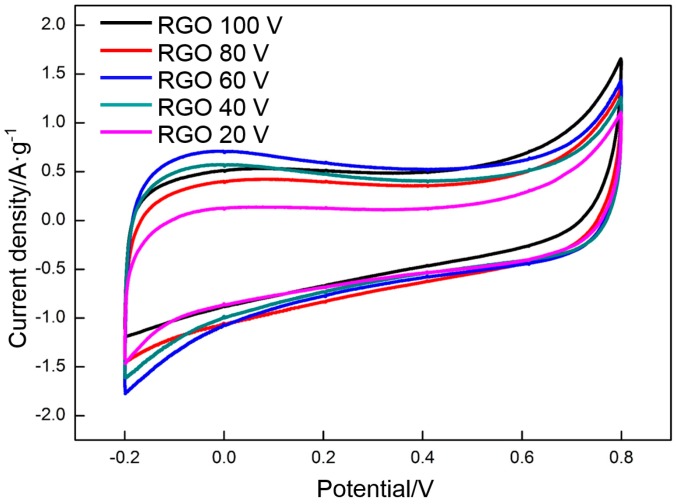
CV curves of RGO at different deposition voltages.

**Figure 12:**
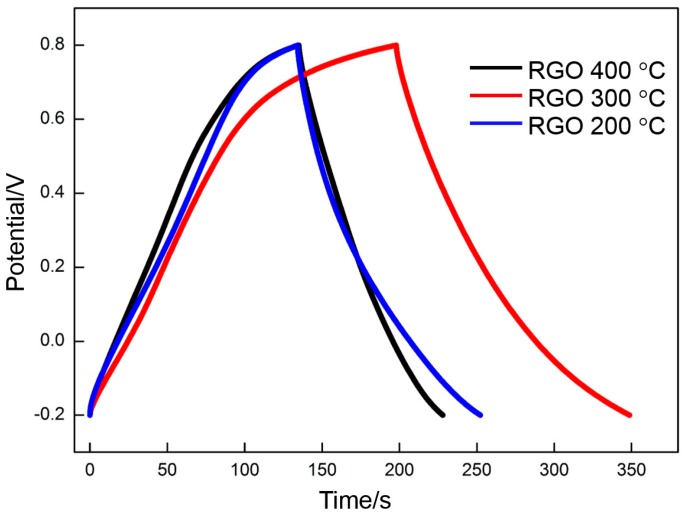
Galvanostatic charge/discharge curves of RGO at different deposition voltages.

GO is deposited at different voltages (20, 40, 60, 80, and 100 V) and reduced at 300 °C, which is the appropriate temperature. The effect of voltage on the specific capacitance of RGO is assessed by CV and galvanostatic charge/charge. Figure 11 and Figure 12 show the CV profiles and the galvanostatic charge/discharge curves of the RGO. The following specific capacitances of the CV measurements are derived: 87 F·g^−1^ (20 V), 121 F·g^−1^ (40 V), 139 F·g^−1^ (60 V), 120 F·g^−1^ (80 V), and 120 F·g^−1^ (100 V). The following specific capacitances of the galvanostatic charge/discharge measurements are derived: 95 F·g^−1^ (20 V), 124 F·g^−1^ (40 V), 151 F·g^−1^ (40 V), 120 F·g^−1^ (40 V), and 118 F·g^−1^ (100 V). The specific capacitance presents a similar change trend with the increasing voltage. The highest specific capacitance is obtained at the deposition voltage of 60 V. The specific capacitance increases with the voltage when the voltage is less than 60 V. However, the specific capacitance decreases with a further increase in voltage. This phenomenon can be explained clearly by the change in the morphology of the deposited GO. The deposited GO layer is comparatively leveled at a comparatively low voltage (<60 V). However, many crumpled patterns are observed around the frameworks of the nickel foam at 60 V, thereby significantly enhancing the specific area of GO. A high specific area implies that many oxygen-containing function groups per unit mass of RGO are exposed to the electrolyte, thereby providing more pseudocapacitance. Meanwhile, the EDLC is also proportional to the specific area. A high specific area can endow the RGO with a high EDLC. The high deposition rate at high voltages (80 and 100 V) promotes the spread of GO from the frameworks to the gaps among the frameworks. Some gaps in the nickel foam surface may be sealed off, thereby separating the RGO inside the nickel foam from the electrolyte. The specific capacitance decreases with the significant reduction of the effective specific area exposed to the electrolyte.

**Figure 13:**
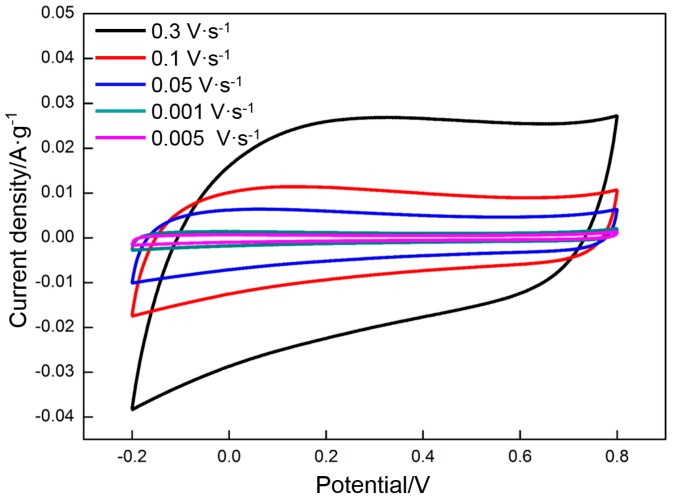
CV curves of RGO at different scan rates.

**Figure 14:**
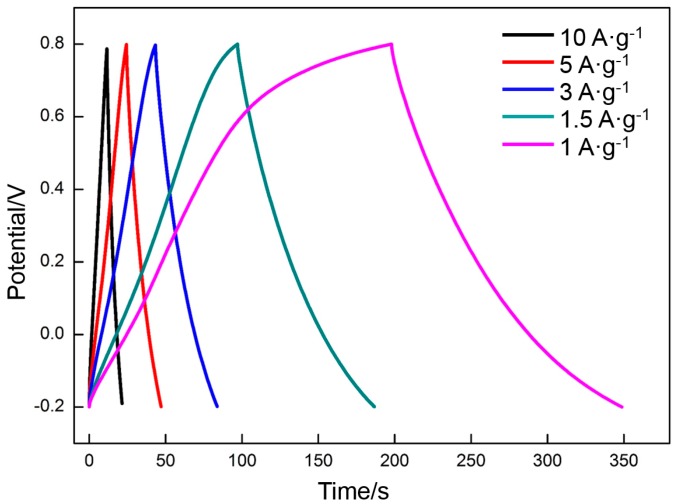
Galvanostatic charge/discharge curves of RGO at different current densities.

The effect of the charge/discharge rate on the specific capacitance of RGO prepared at the appropriate deposition voltage (60 V) and thermal reduction temperature (300 °C) was investigated. Figure 13 shows the CV profiles of RGO at different scan rates from 0.005 to 0.3 V·s^−1^. Rectangular curves are still obtained at a high scan rate (0.3 V·s^−1^), thereby indicating that RGO has excellent capacitance properties. Using Equation (2), the following specific capacitances of RGO at different scan rates are derived: 139 F·g^−1^ (0.005 V·s^−1^), 130 F·g^−1^ (0.01 V·s^−1^), 109 F·g^−1^ (0.05 V·s^−1^), 98 F·g^−1^ (0.1 V·s^−1^), and 77 F·g^−1^ (0.3 V·s^−1^). The specific capacitances decrease with the increase in scan rate. However, the charge/discharge time is greatly shortened. The charge/discharge durations are 400 s (0.005 V·s^−1^), 200 s (0.01 V·s^−1^), 40 s (0.05 V·s^−1^), 20 s (0.1 V·s^−1^), and 7 s (0.3 V·s^−1^), respectively. The galvanostatic charge/discharge curves obtained at different current densities (1 A·g^−1^ to 10 A·g^−1^) also exhibit a symmetrical triangular structure (Figure 14). The specific capacitances of the RGO at different current densities are 151 F·g^−1^ (1 A·g^−1^), 135 F·g^−1^ (1.5 A·g^−1^), 121 F·g^−1^ (3 A·g^−1^), 114 F·g^−1^ (5 A·g^−1^), and 100 F·g^−1^ (10 A·g^−1^). The charge/discharge times are 151, 90, 40, 23, and 10 s, respectively. The results are consistent with those in CV. The specific capacitances present a decreasing incline with the increasing charge/discharge rate, which is related to the diffusion of ions in the electrolyte. Both EDLC and pseudocapacitance depend on the diffusion of ions. The diffusion rate of ions falls behind the charge/discharge rate when the latter is too high, thereby decreasing the specific capacitance.

As mentioned above, some carbon materials had been prepared for the electro material of the supercapacitor [5,6,7,8,9,10,11,12]. As shown in Table 1, their specific capacitance is usually 70–160 F·g^−1^. Compared with the previously reported specific capacitance of the carbon materials, the RGO electrode prepared in this work shows a high specific capacitance, which can be easily found in Table 1. Moreover, the preparation of the electrode is also simpler than those reported.

**Figure 15:**
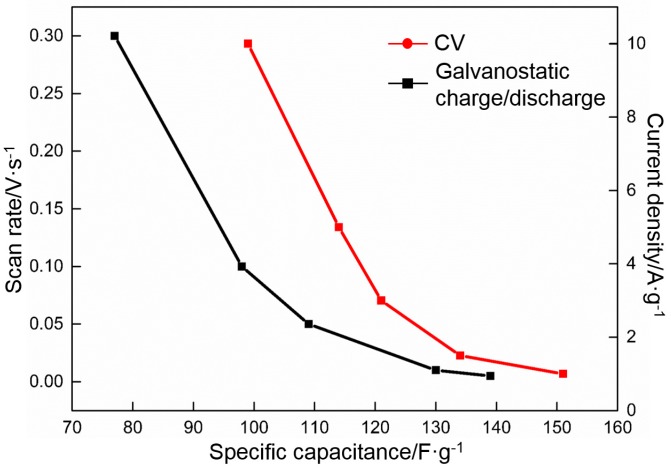
Specific capacitance of RGO as a function of scan rate and current density.

Figure 15 shows that the specific capacitance of RGO is a function of the charge/discharge rate. RGO retains approximately 55% of the value obtained in 0.005 V·s^−1^ as the scan rate is increased from 0.005 to 0.3 V·s^−1^ and approximately 66% of the values obtained in 1 A·g^−1^ when the current density is increased from 1 to 10 A·g^−1^. Therefore, RGO exhibits an outstanding rate performance.

**Figure 16:**
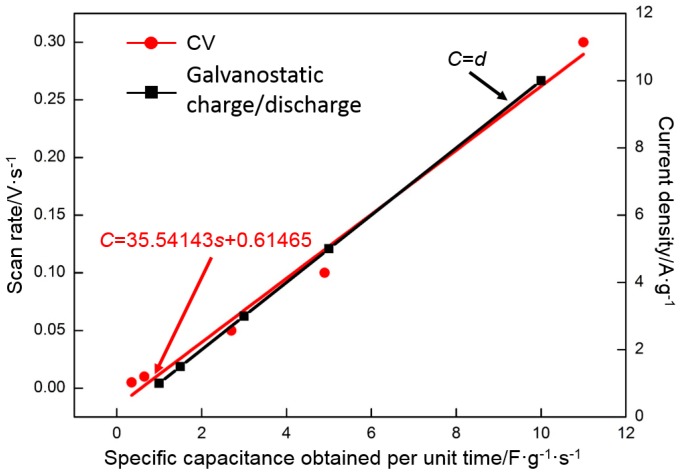
Specific capacitance of RGO obtained per unit time as a function of the scan rate and current density.

The charge/discharge rate is an important performance indicator of a supercapacitor and determines its speed of storing or releasing a certain amount of energy. In addition to the capacity retention ratio mentioned above, the rate performance can be characterized by an index-specific capacitance obtained per unit time (F·g^−1^·s^−1^). Figure 16 illustrates the relationship between the index and the charge/discharge rate. The CV data are processed by the least squares method, and the fitting result is as follows:

*C* = 35.54143*s* + 0.61465 (7)

in which *s* is the scan rate (V·s^−1^) and *C* is specific capacitance obtained per unit time (F·g^−1^·s^−1^).

The galvanostatic charge/discharge data are also processed by the least squares method, and the fitting result is as follows:

*C* = *d* (8)

in which *d* is the current density (A·g^−1^) and *C* is specific capacitance obtained per unit time (F·g^−1^·s^−1^).

Along with the increase in the charge/discharge rate, the specific capacitance obtained per unit time approximately presents a linear increase. A high charge/discharge efficiency can be obtained with a high charge/discharge rate. The specific capacitance of 0.3 V·s^−1^ is half that of 0.005 V·s^−1^; however, its specific capacitance obtained per unit time of charge/discharge is 31 times that of 0.005 V·s^−1^. Similarly, the specific capacitance of 10 A·g^−1^ is two thirds that of 1 A·g^−1^; however, the specific capacitance obtained per unit time is 10 times that of 1 A·g^−1^. The results further show that RGO presents an excellent rate performance and can store or release a large amount of energy in a short time. A high charge/discharge ratio contributes to the extensive application of RGO in the industry.

**Figure 17:**
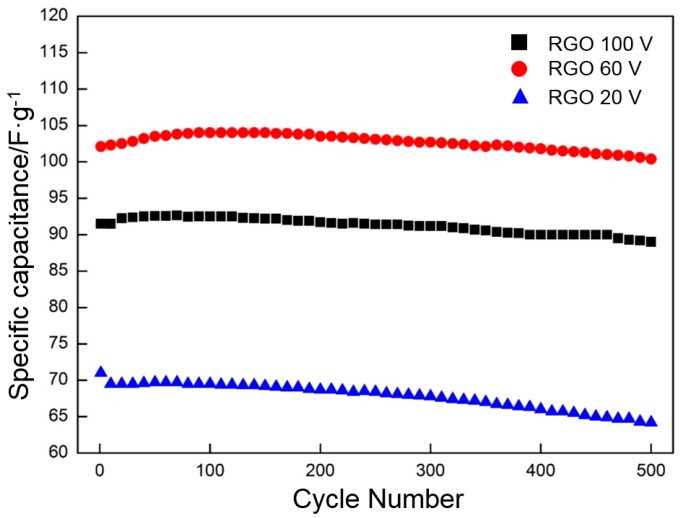
Cycling test for RGO (deposited at different deposition voltages and reduced at 300 °C) at a scan rate of 0.05 V·s^−1^ up to 500 cycles.

Cycling stability is another important supercapacitor parameter. The cycle performance of RGO (deposited at 20, 60, and 100 V and reduced at 300 °C) is evaluated by CV for 500 cycles at 0.05 V·s^−1^ (Figure 17). The specific capacitances of RGO deposited at 20 and 100 V gradually decline with the increasing cycling number. After 500 cycles, the specific capacitance remains at approximately 90% (20 V) and 97% (100 V) of the initial value. The specific capacitance of the RGO deposited at 60 V slightly increases from the initial 102.1 to 104 F·g^−1^ after 100 cycles. This response may be because more oxygen-containing functional groups are activated during the cycling process than at other stages [52]. Then, the specific capacitance begins to decrease. After 500 cycles, approximately 98% of the specific capacitance is retained, thereby demonstrating the excellent cycle stability of RGO.

## 4. Conclusions

1. Deposition voltage has a significant effect on the morphologies of deposited GO. At a low deposition voltage (less than 60 V), the frameworks in the nickel foam are not completely covered with GO. Some holes around the frameworks are covered at a high deposition voltage (higher than 60 V). The frameworks in the nickel foam are only covered with a uniform dense deposition layer at the deposition voltage of 60 V.
2. GO is reduced in a very narrow temperature range of 160 to 240 °C. The thermal reduction of GO at 300 °C contributes to the enhancement of the EDLC. At the same time, several of the oxygen-containing functional groups in GO are maintained, which is beneficial to the increase in pseudocapacitance.
3. The specific capacitance of RGO is closely related to the deposition voltage and the thermal reduction temperature. The RGO deposited at 60 V and thermally reduced at 300 °C exhibits the highest specific capacitance of all. The specific capacitances calculated by using CV and galvanostatic charge/discharge are 139 and 151 F·g^−1^, respectively.
4. The specific capacitance of RGO is also connected with the charge/discharge rate. Along with the increase in charge/discharge rate, the specific capacitance presents the decreasing trend, accompanied with the increase in charge/discharge time (CV: 0.005 V·s^−1^, 139 F·g^−1^/400 s; 0.01 V·s^−1^, 130 F·g^−1^/200 s; 0.05 V·s^−1^, 109 F·g^−1^/40 s; 0.1 V·s^−1^, 98 F·g^−1^/20 s; 0.3 V·s^−1^, 77 F·g^−1^/6.7 s. Galvanostatic charge/discharge: 1 A·g^−1^, 151 F·g^−1^/151 s; 1.5 A·g^−1^, 135 F·g^−1^/90 s; 3 A·g^−1^, 121 F·g^−1^/40.4 s; 5 A·g^−1^, 114 F·g^−1^/20.8 s; 10 A·g^−1^ 100 F·g^−1^/10 s). The specific capacitance obtained per unit time (F·g^−1^·s^−1^) is used to characterize the charge/discharge efficiency. The index is increased linearly with the charge/discharge rate (CV: 0.005 V·s^−1^, 0.35 F·g^−1^·s^−1^; 0.01 V·s^−1^, 0.65 F·g^−1^·s^−1^; 0.05 V·s^−1^, 2.7 F·g^−1^·s^−1^; 0.1 V·s^−1^, 4.9 F·g^−1^·s^−1^; 0.3 V·s^−1^, 11 F·g^−1^·s^−1^. Galvanostatic charge/discharge: 1 A·g^−1^, 1 F·g^−1^·s^−1^; 1.5 A·g^−1^, 1.5 F·g^−1^·s^−1^; 3 A·g^−1^, 3 F·g^−1^·s^−1^; 5 A·g^−1^, 5 F·g^−1^·s^−1^; 10 A·g^−1^, 10 F·g^−1^·s^−1^).
5. The RGO deposited at 60 V and thermally reduced at 300 °C exhibits an excellent cycle stability and maintains approximately 98% of the initial specific capacitance after 500 cycles.

## Figures and Tables

**Table 1:** Comparison of specific capacitance between this work and reported references.

Electrode	Specific Capacitance (F·g^−1^)	Current Density or Scan Rate	References
RGO	151	1 A·g^−1^	This work
ACs	145	20 A·g^−1^	[5]
ACs	155	0.05 A·g^−1^	[6]
ACs	160	0.1 A·g^−1^	[7]
OHMMC	146	0.1 A·g^−1^	[8]
MCNAs	152	0.1 A·g^−1^	[9]
3D HOPC	73.4	3 mV·s^−1^	[10]
CNTs	180	100 mV·s^−1^	[11]
SWNT	114	1 mV·s^−1^	[12]
